# Recent Progress in Advanced Polyester Elastomers for Tissue Engineering and Bioelectronics

**DOI:** 10.3390/molecules28248025

**Published:** 2023-12-09

**Authors:** Yawei Zhao, Wen Zhong

**Affiliations:** 1 Department of Biosystems Engineering, University of Manitoba, Winnipeg, MB R3T 2N2, Canada; zhaoy13@myumanitoba.ca; 2 Department of Medical Microbiology, University of Manitoba, Winnipeg, MB R3T 2N2, Canada

**Keywords:** polyester, elastomers, tissue engineering, bioelectronics

## Abstract

Polyester elastomers are highly flexible and elastic materials that have demonstrated considerable potential in various biomedical applications including cardiac, vascular, neural, and bone tissue engineering and bioelectronics. Polyesters are desirable candidates for future commercial implants due to their biocompatibility, biodegradability, tunable mechanical properties, and facile synthesis and fabrication methods. The incorporation of bioactive components further improves the therapeutic effects of polyester elastomers in biomedical applications. In this review, novel structural modification methods that contribute to outstanding mechanical behaviors of polyester elastomers are discussed. Recent advances in the application of polyester elastomers in tissue engineering and bioelectronics are outlined and analyzed. A prospective of the future research and development on polyester elastomers is also provided.

## 1. Introduction

Polyesters are polymers formed through monomer(s) linked by ester bonds. Naturally occurring esters are present in the human body, such as fatty acids [1]. Most polyesters show hydrophobic properties attributed to their long alkyl chains, and their mechanical performance can be fine tuned by modifying the polymer chain structure or adjusting the ratios of monomers [2]. An important characteristic of polyesters is their innate biodegradability. When polyesters are implanted into the human body, their degradation can be triggered through the breaking down of ester bonds by esterases or/and hydrolysis. This in vivo degradation process plays a crucial role in the tissue engineering applications of polyesters, facilitating the gradual transfer of mechanical burden and biofunctions from the degrading scaffolds to the regenerated tissue [3,4].

Many polyesters exhibit elasticity, allowing the polymers to regain their original shape after deformation. Elastomers are a type of polymer characterized by its viscoelastic properties, relatively low Young’s modulus, and high breaking elongation [5]. While many materials possess an elastic region, the term “elastomer” typically is used to describe a material that features a highly expansive elastic region with a large strain, typically above a few hundred percent [6]. The polyester elastomers can be categorized into physically crosslinked and chemically crosslinked polyesters [7]. Physically crosslinked polyesters are crosslinked with physical interactions including crystalline regions, hydrogen bonds, and dipolar forces. The physically crosslinked regions, generally including the crystalline region [8,9,10] and reinforcing nanofillers [11], provide crosslinking sites and rigidity. The amorphous regions in elastomers, on the other hand, contribute to flexibility. Typical physically crosslinked polyesters include poly(ε-caprolactone) (PCL), poly(glycolic acid) (PGA), poly(lactic acid) (PLA), and their copolymers. Chemically crosslinked polyesters have their polymer chains interconnected through covalent bonds, typically formed by multifunctional monomers. For instance, citric acid is a monomer with three carboxylic acid groups that can react with alcohol compounds with two hydroxy groups to synthesize poly(diol citrate)s (PDCs). To match the mechanical properties of human tissues, the polyester elastomers can be tailored to the MPa ranges in strength by tuning the monomers, molecular weight, processing conditions or structural design of the polymers, and therefore expand their biomedical applications in tissue engineering, medical implants, and drug delivery [12].

In recent years, polyesters have been playing an increasing role in biomedical research and applications. Polyester elastomers are biocompatible, biodegradable, and reproducible, making them excellent alternatives of allografts (Table 1). Advanced methods and technologies have been developed for polyester elastomers fabricated with various crosslinking strategies and designed for a variety of properties and functionalities for different biomedical applications. In this review, recent progress in the development and evaluation of polyester elastomers will be summarized, and their applications in biomedical fields including cardiac, vascular, neural, and bone tissue engineering and bioelectronics will be discussed. Future perspectives in this area will also be provided. 

## 2. Synthetic Pathways and Functionalization of Polyester Elastomers

### 2.1. Synthetic Pathways of Representative Polyesters

#### 2.1.1. Physically Crosslinked Polyester Elastomers

**Figure 1:**
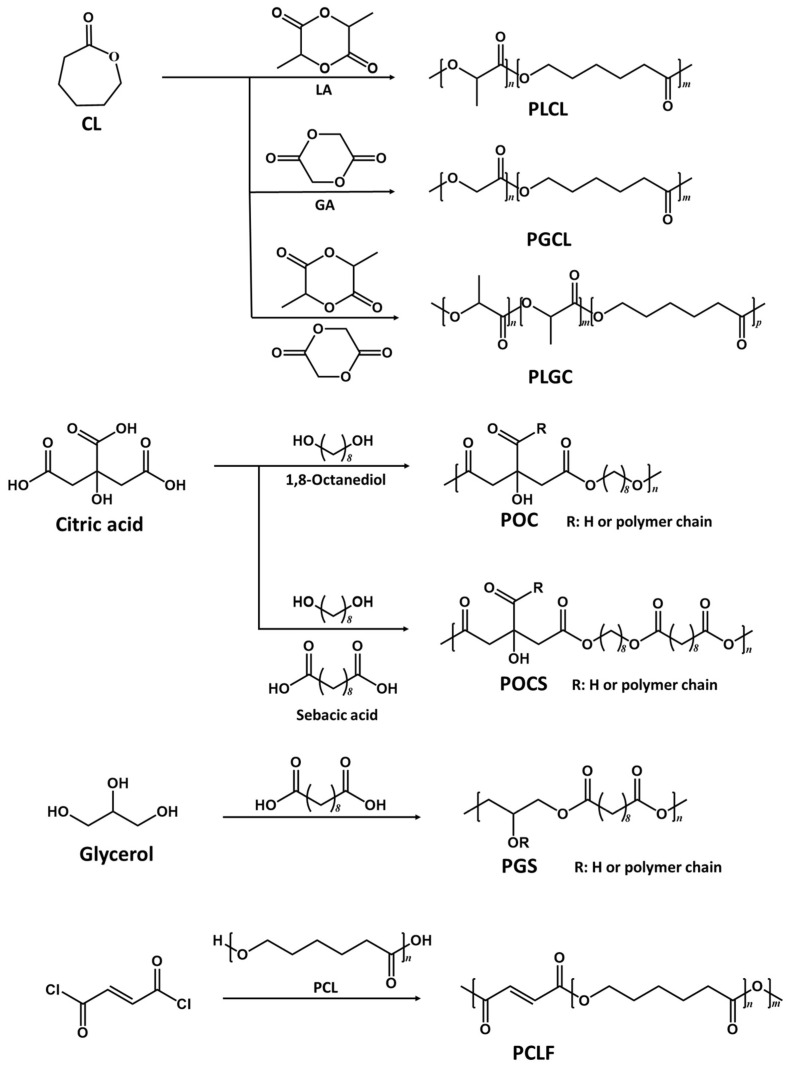
Synthetic routes of representative polyesters. CL: ε-caprolactone, LA: L-lactide, PLCL: poly(L-lactide-*co*-ε-caprolactone), GA: glycolic acid, PGCL: poly(lactic-*co*-glycolic acid), PLGC: poly(lactide-*co*-glycolide-*co*-caprolactone), POC: poly(octanediol-*co*-citric acid), PGS: poly(glycerol sebacate), PCLF: poly(caprolactone fumarate).

Typical physically crosslinked polyesters include PLA, PCL, PGA, and their copolymers, which are attractive synthetic polymers in tissue engineering due to their biocompatibility and biodegradability. They have been approved by the FDA for biomedical applications [32,33,34,35]. Their copolymers are thermoplastic elastic biomaterials [36] (Figure 1). These polymers can be polymerized through ring-opening reactions using catalyst stannous(II)octoate [Sn(Oct)_2_] [37]. By optimizing the monomer ratios in the copolymerization process, fine tuning of the mechanical properties and degradation profiles of these materials can be achieved.

#### 2.1.2. Chemically Crosslinked Polyester Elastomers

The representative chemically crosslinked polyester elastomers include poly(polyol sebacate) (PPS) and PDC (Figure 1). In the PPS family, the polyol monomers can be biomass-derived monomers, such as glycerol, iso-sorbitol, maltitol, erythritol, and xylitol [38]. Among them, poly(glycerol sebacate) (PGS) has been a material attracting extensive research. Many aliphatic diols have been used to synthesize PDC, and the most studied is 1,8-octanediol [39]. Polymerization processes for PPS and PDC are similar and typically involve two steps. Firstly, the prepolymers are synthesized by thermal polycondensation of the esterification reaction between -OH and -COOH groups. The reaction temperature and time vary based on the specific type of monomers involved. Secondly, post polymerization takes several days to form the elastomers at a desired condition due to the covalent crosslinking between unreacted -OH and -COOH groups [21,40,41].

### 2.2. Route of Degradation

In vivo biodegradability of polyesters is of utmost importance for their biomedical applications. Extensive research has been conducted to examine their degradation pathways and identify the most suitable materials for specific biomedical applications. The degradation mechanisms include surface erosion and bulk erosion. Surface-eroding polyesters involve PGS, poly(trimethylene carbonate) (PTMC), poly(ethylene carbonate), and poly(anhydride) [42]. The degradation primarily occurs at the material surfaces, and the mass loss and dimensional reduction in these materials are related to their surface area. Consequently, the integrity of these materials is preserved during the degradation process, and their properties will remain unchanged until they have fulfilled their intended treatment purpose [43]. Bulk-eroding polyesters mainly include aliphatic polyesters and PDC. The degradation of these polyester elastomers occurs within the bulk materials, resulting in a decrease in molecular weight. During the initial stages, the mass of the materials remains relatively stable, but there are significant changes in their properties. As the molecular weight gradually decreases and reaches a critically low value, the materials ultimately collapse or disintegrate, resulting in a quick release of the degradation products, which can alter the local microenvironment or induce tissue response [44]. If the bulk erosion elastomer is designed to deliver bioactive agents, its sudden collapse may cause fast drug release, which may become a risk to patients. In contrast, the surface-eroding scaffolds can provide a relatively constant drug administration; therefore, they are preferable in biomedical applications. 

### 2.3. Functionalization of Polyesters

Elastomers offer an essential elastic recoil capacity, which is vital for preserving the functions of such natural tissues as the heart, lungs, blood vessels, and skin. In some randomly coiled polymers such as elastin and silicone, polymer chains are linked via covalent bonds to form elastomers [45]. Weak bonds perform similar tasks in polyurethanes, polyamide, and polyvinyl chloride [46]. Different polymers may have specific structures/crosslinking bonds to form networks with a variety of physical and chemical properties. This complexity in elastomer design may constrain the versatility and the scope of properties achievable in the resulting materials [47]. 

Functionalization has been explored to obtain elastomers with a wide variety of properties. For polyester elastomers, such modification methods as urethane doping, alkene groups modification, and silicon doping, have been widely studied [48,49]. Polyesters incorporated with urethane monomers have been demonstrated to enhance the mechanical properties of elastomers while preserving their biocompatibility. Acrylated polyesters retain their biodegradability and biocompatibility, and are endowed with photo curability, contributing to their 3D printability. For example, itaconate was introduced into the poly(octanediol-co-citric acid) (POC) backbone as an unsaturated component [50], contributing to controlled and quick curing of the elastomers. These elastomers were gel-like polymers which can be 3D printed into various shapes depending on their applications. By adjusting reaction times and molar ratios of monomers, materials with a wide range of elasticity (Young’s modulus in the range of 36–1476 kPa) were synthesized, indicating the mechanical tunability of materials. These poly(itaconate-*co*-citrate-*co*-octanediol) (PICO) elastomers were further applied as cardiac tissue patches to provide the necessary elastomeric support and result in visible tissue organization and viability. 

**Figure 2:**
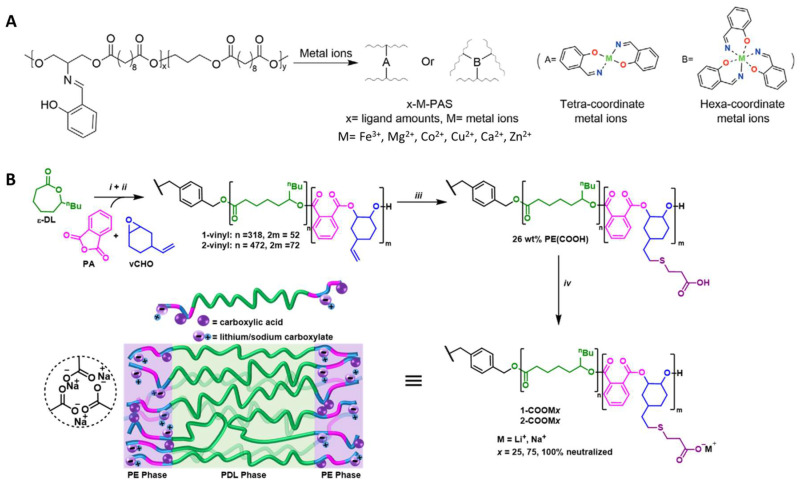
Functionalization of polyesters. (**A**) Chelation crosslinking of polyester elastomers. Reproduced with permission from Ref. [47]. Copyright 2020 Wiley. (**B**) Ionic interactions of polyester elastomers [59]. (i) Catalyzation by catalyzed by LZnMg(C_6_F_5_)_2_, (ii) addition of raw materials, (iii) thiol —ene reaction, (iv) neutralization of the carboxylic acid with LiOH or NaOH. Copyright 2022 American Chemical Society.

Recent interests have grown towards dynamic covalent coordination bonds, which provide a new way to enrich the range of mechanical properties and to introduce self-healing properties, which enable molecular binding between separated or damaged interfaces [51,52,53,54,55,56,57]. The coordination ligands can bind different kinds of metal ions and form different coordination bonds, imparting versatility to the polymer networks. The strengths of different coordination bonds are different, leading to various mechanical properties and biodegradability of the resultant polymers. Chen et al. [47] developed polyester elastomers from monomers sebacic acid and 1,3-propanediol with the Schiff base coordination bond ligand (2-[[(2-hydroxyphenyl)methylene]amino]-1,3-propanediol (HPA) (Figure 2A). Biologically relevant metal ions such as Mg^2+^, Ca^2+^, Fe^3+^, Cu^2+^, Zn^2+^, and Co^2+^ were mixed with the polymers using various ratios of metal to ligand and ligand density to provide materials with a wide range of mechanical properties. The biocompatibility of the elastomers matched that of PCL and showed promising potential for soft tissue regeneration. Guo et al. [58] designed PCL-based elastomers with dynamic coordination bonds that contributed to a toughness of 372 MJ m^−3^ and a significant fracture energy of 646 kJ m^−2^. Protocatechualdehyde (PA) as a chain extender and Fe^3+^ were used in iron-catechol coordination. The obtained elastomers were biocompatible and can be applied as surgical sutures to improve wound healing.

Non-covalent interactions were also used to improve polyester elastomers in elasticity and robustness [60]. Introducing hydrogen bonds into elastomers is an effective way to tune their properties [61,62]. For example, 2-Ureido-4[1H]-pyrimidone (UPy), a supramolecular assembly, was widely studied recently because it can improve the properties of elastomers by the formation of quadruple hydrogen bonding [63]. PCL functionalized with UPy groups has been shown to enhance elasticity due to the reversible intermolecular hydrogen bonds that help with energy dissipation [53]. Furthermore, some Upy-modified bioactive molecules can bind to the UPy moieties in elastomers, providing an additional function to the scaffold. Gregory et al. [59] introduced ionic interactions into poly(ε-decalactone) (PDL)-based polyester elastomers. Lithium or sodium ions were added to polyesters terminated with -COOH groups to form carboxylates to improve the tensile strength and elasticity of ionized elastomers (Figure 2B).

## 3. Biomedical Applications of Polyester Elastomers

### 3.1. Cardiac Tissue Engineering

Cardiac tissue engineering (CTE) has been an important branch of tissue engineering (TE) with the objective of creating a cell-scaffold structure that facilitates repairing of cardiac tissues [64]. Myocardial tissues exhibit an intricate architecture, comprising various cell types, including cardiomyocytes, fibroblasts, smooth muscle cells (SMCs), endothelial cells (ECs), and extracellular matrix (ECM) that comprise fibrin, collagen, and elastin. Both the cells and the ECM contribute to the elastomeric mechanical characteristics and physiological functions of the heart [65]. The ECM is crucial in facilitating cell interconnection, transmitting signals, and maintaining the tissue’s mechanical properties [66]. The end-diastolic Young’s modulus of typical human myocardial tissue is 0.2–0.5 MPa and tensile strength is 3–15 kPa, demonstrating its high elasticity [67]. Various approaches have been reported to promote myocardial tissue repair, including efforts to replicate the microenvironment of the native myocardium, stimulate the recruitment and division of cardiac cells, improve tissue vascularization, and regulate the secretion of factors associated with repair [48,68].

**Figure 3:**
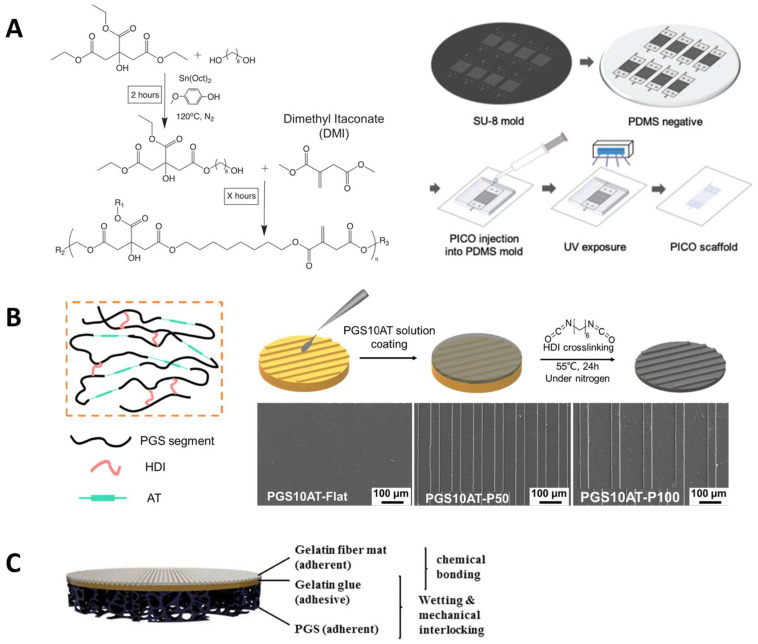
Cardiac tissue engineering applications. (**A**) One-pot synthesis and fabrication of PICO elastomers. Reproduced with permission from Ref. [50]. Copyright 2019 Wiley. (**B**) Micropatterned PGS-*co*-aniline elastomer for cardiac tissue engineering. Reproduced with permission from Ref. [75]. Copyright 2019 Elsevier. (**C**) Layered cardiac patches based on PGS and gelatin [76]. Copyright 2020 Wiley.

Various strategies have been reported to develop polyester scaffolds with matching mechanical properties to that of host myocardial tissues. Polyesters can be blended with other polymers, inorganic fillers or modified with unsaturated bonds to obtain suitable mechanical properties and biodegradation rates. For example, PGS was combined with a multiblock thermoplastic polymer poly (butylene succinate-butylene dilinoleate) (PBS-DLA) to obtain a material with desired properties to be applied in cardiac regeneration [69]. The resulting elastomers showed higher mechanical property: the storage modulus E’ was nearly doubled from 23 ± 11 MPa to 39 ± 7 MPa when the PBS-DLA contents were increased from 30% to 60%. And the composite patches showed slower degradation than PGS, which were more suitable for use in cardiac patches. Increased C2C12 cell viability was observed on cardiac patches with higher PBS-DLA content. Huyer et al. designed unsaturated polyester elastomers (poly(itaconate-co-citrate-co-octanediol) (PICO) and employed itaconic acid as a co-monomer in the copolymerization of a POC (Figure 3A). The PICO elastomers displayed an adjustable elasticity within a range of 36–1476 kPa by tuning the crosslinking density and were shown to support the organization and viability of cardiac tissue [50]. To make the unsaturated polyester better attached to the cardiac tissue, Bannerman et al. reported an elastomer patch with satisfying adhesive strength by introducing dopamine (DA) into poly(ocatamethylene maleate(anyhydride)citrate) (POMaC) polymers. The biocompatible adhesive patches showed good adhesive strength (~0.43 N/cm^2^) to cardiac tissue, which was better than that of POMaC and fibrin glue (~0.11 N/cm^2^ and ~0.16 N/cm^2^) due to the interaction between DA and tissue. The elastic modulus of the patch was 51.4 ± 4.1 kPa, similar to the cardiac tissue [70]. 

Moreover, blending with other bioactive polymers or inorganic fillers can endow scaffold therapy effects and better tissue recovery [71]. PGS was mixed with polypyrrole (PPy) to fabricate a semiconductive polymer film as cardiac repairing patches. The introduction of conductive components was found to help with cardiac tissue maturity because cardiac tissue has an electromechanical property [72]. Some recent patents described the structural design of polyester elastomers for CTE. For example, 1,2,4-butanetricarboxylic acid was polycondensed with maliec anhydide and 1,8-octanediol to functionalize the polymer with UV crosslinked property. This material was molded and UV crosslinked into mesh structures or porous scaffolds with customized shapes/sizes. The elastomeric properties could be tuned by optimizing monomer ratios to match those of the myocardium. These elastomers also showed approparaite degradation behaviors as a scaffold support for potential implants [73]. In another patent, the hexamethylene diisocyanate (HDI) crosslinked PGS (PGSU) elastomers were developed to mimic the viscoelastic properties of tissue [74]. 

Different fabrication techniques have been explored to generate porous scaffold with appropriate thickness for cardiac cellular proliferation. Solvent casting, electrospinning, micropatterning, and 3D printing have been the most used fabrication methods of CET scaffolds [77]. Hu et al. created the PGS-*co*-aniline elastomeric scaffold with electroactivity using aniline as the conductive component [75] (Figure 3B). These films exhibited electroactive characteristics and an appropriate elastic modulus that could support heart tissue regeneration; Young’s modulus was 20.2 ± 3.6 MPa and the elongation varied from 27% to 141%. The scaffolds were biodegradable and the micropatterning fabrication method enhanced the viability and proliferation of cardiomyoblast-derived H9c2 cells of rat, as well as ridge surface guided cardiomyocytes’ alignment and elongation. The biocompatibility test for the films proved a similar biological response to control the PGS group with good biocompatibility. Yang et al. designed an elastic cardiac scaffold using mixtures of biocompatible PCL and PGS and employing 3D printing technology to obtain the construction with regular patterned filaments and interconnected micropores [78]. The scaffold showed superior mechanical properties and could be tailored to custom shapes through 3D printing. Young’s modulus of PCL-PGS was 748.5 ± 21.0 kPa, the tensile strength was 748.5 ± 21.0 kPa, and the elongation was 57.3 ± 1.3%. In an infarcted myocardium model, the implanted PCL-PGS scaffold was found to promote heart functions. A flexible microneedle array was fabricated by solvent casting using PLGA, PCL or a combination of these two polymers [79]. This microneedle array showed flexible mechanical properties, facilitating safe wearability for patients. The drug delivery capacity was also promoted due to the higher contact area.

Polyesters have also been combined with natural polymers. Natural polymers provide important ligands for cell adhesion and proliferation (e.g., arginine-glycine-aspartic acid (RGD) tri-peptide unit that is present in almost all ECM protein fibronectin) [80,81,82,83]. The primary degradation products of natural polymers are harmless, and therefore trigger a minimal immune response [70]. Ruther et al. designed a layered scaffold based on porous PGS substrates. A top layer of electrospun gelatin fiber mat was deposited on the substrates, glued by a middle layer of gelatin [76]. Adhesion and degradation tests suggested that gelatin gluing, which bonded all the components by forming chemical bonds, showed better results even after being kept in the phosphate buffered saline for two weeks: the crosslinked gelatin was relatively stable and the PGS substrates were still adhered together. These results demonstrated that the layered cardiac patches composed from porous PGS substrates and electrospun biopolymer fibers had potential in CTE (Figure 3C). 

In addition to preformed scaffolds, injectable polyesters have been developed, simplifying the material application process. Hamada et al. reported a controlled release strategy to deliver extracellular vesicles (EVs) using a photocurable polyester to cardiac tissue, aimed at improving the muscle damage [84]. The PGSA-g-EG mixture was injected to the affected myocardium and photo crosslinked with an LED light (405 nm) in situ. The authors proved that this composition did not impact the bioactivity of EV and could control the release of EV for two weeks due to the in vivo surface erosion degradation. In vitro cytotoxicity tests of PGSA-g-EG using H9c2 rat cardiac myoblasts and in vivo biocompatibility experiments using male Wistar rats demonstrated no toxicity and no morphologic alterations when the polymer was applied on tissue.

### 3.2. Vascular Tissue Engineering

Vascular tissue comprises cells, proteins, and ECM that form a tubular layered structure [85]. Typical vascular tissue exhibited viscoelastic mechanical properties, its tensile strength is about 4.3 MPa, and the burst pressure is up to 3000 mmHg. A special biological function of vascular tissue is that they can prevent platelet adhesion [86]. Several strategies have been reported to promote vascular tissue repair, including mimicking the natural vascular tissue structures, preventing platelet adhesion, improving endothelialization, delivery, and release of bioactive materials [87,88]. Regarding polyester elastomer materials, braiding, electrospinning, salt leaching, and 3D printing have been explored for vascular TE applications. 

**Figure 4:**
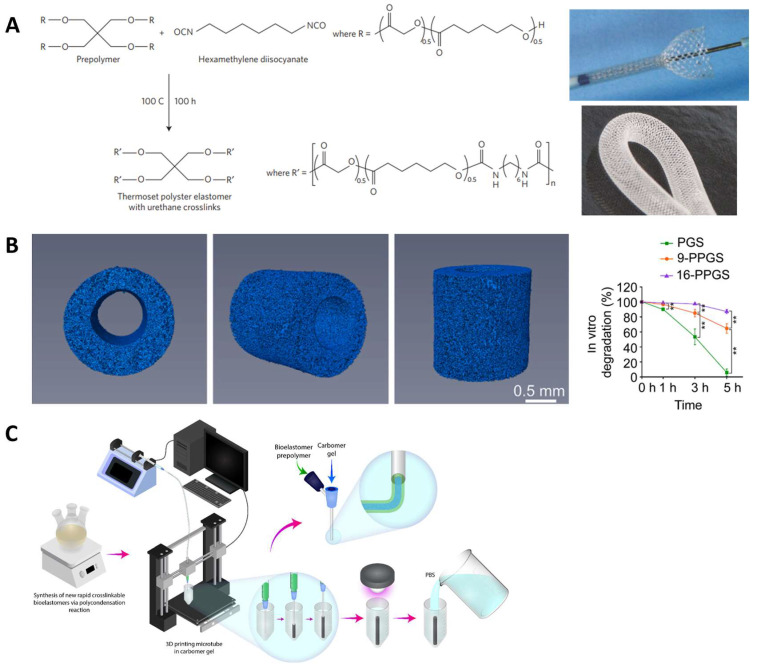
Vascular tissue engineering applications. (**A**) Synthesis of the PGCL elastomer coating and the braided implants for porcine femoral artery. Reproduced with permission from Ref. [91]. Copyright 2018 Springer Nature. (**B**) PGS derivative-based scaffolds with slow degradation to improve vascular graft remodeling. The 3D images of grafts and in vivo degradation. ** indicates *p* < 0.01, compared between two groups. Reproduced with permission from Ref. [93]. Copyright 2020 Elsevier. (**C**) Three-dimensional printing of vascular tubes using bioelastomer prepolymers PITCO [95]. Copyright 2020 American Chemical Society.

For arterial tissue engineering, strong, bioresorbable scaffolds are needed to provide temporary strength, namely, to hold an expanded vessel and to resist vessel recoil until the healing process is completed [89,90]. To achieve appropriate bioabsorbability and mechanical properties, Sharma et al. [91] designed scaffolds from PGA and L-PLGA fibers that were braided. Then, the braided scaffold was coated with four-arm poly(glycolide-co-caprolactone) (PGCL) elastomer which was crosslinked by HDI (Figure 4A). The elastomer-coated scaffolds showed higher compression strength and elasticity than the scaffolds without coating (~700 mmHg vs. ~100 mmHg), measured by the radical stiffness (RRF). Their expansion properties were also improved and similar to those of metallic stents, meeting the expanding requirement of vessel treatment, the data were ~150 mmHg vs. ~30 mmHg, measured by the chronic outward force (COF). Mechanical properties of the elastomers can be adjusted by changing their branching structure, crosslinking density, and molecular weight. These scaffolds showed a promising application as vessel implants. Their biocompatibility was evaluated by an ovine model, in which inflammation was found to be lower than the moderate amount, demonstrating acceptable biocompatibility as vascular implants. Zhao et al. [90] also reported a bioresorbable stent comprising poly(p-dioxanone) (PPDO) monofilaments and polycaprolactone/poly(p-dioxanone) (PCL/PPDO) core-shell composite yarns via braiding. The degradation profile of the bioresorbable stent was optimized to match the vascular remodeling process and to promote healing. Self-expanding stents made of polyester were also described in some patents. For example, one design contains an inner stent made from bioabsorbable metal Nitinol, and an outer stent that comprises PLLA or PGA [92]. The outer layer was configured to be absorbed in vivo, allowing for the inner stent to expand to a larger diameter. More recently, Fu et al. [93] reported PGS-based porous vascular grafts using the salt-leaching method (Figure 4B). They introduced palmitic acid into PGS (palmitic acid-PGS, PPGS) to slow the degradation of the materials and to be synchronous with in vivo regeneration of common carotid artery (CCA). The PPGS showed lower Young’s modulus and larger water contact angles than PGS. The degradation of the developed grafts varied from 4 to 12 weeks, which was longer than that of PGS, which degraded in approximately 2 weeks [94]. When implanted into rat common carotid arteries, better vascular conduits regeneration results of PPGS also demonstrated the slow degradation modification to match the regeneration rate, which can improve the overall performance of vascular grafts during their transformation into autologous vascular conduits.

POC-based elastomers have been the subject of extensive research in vascular TE [45]. In addition to good mechanical properties and biocompatibility, POC elastomers show less platelet adhesion and clotting, minimal hemolysis, and appropriate protein adsorption, indicating their excellent hemocompatibility [96]. Gregory et al. [97] fabricated all-trans retinoic acid(atRA)-POC (POCR)-coated expanded polytetrafluoroethylene (ePTFE) (POCR-ePTFE) vascular graft, which promoted endothelialization. The performance of this material combined previous reported advantages of POCR and POC-ePTFE [98,99]. The atRA was applied to inhibit intimal hyperplasia and accelerate reendothelialization of vascular; ePTFE was utilized as a scaffold material. The atRA-POC-ePTFE performed better in vascular regeneration than the previous atRA-ePTFE as shown in the in vivo experiments. Urethan doping [100] and unsaturated polyester doping [101] have been used to tune the mechanical properties of POC to fit vessel applications. For example, maleic anhydride [102] and itaconic acid [50] were incorporated into the POC backbone to fabricate vascular scaffolds. Montgomery et al. [102] studied poly(octamethylenemaleate (anhydride) citrate (POMaC) with different monomer ratios to tune the elasticity, which ranged from 5.4 kPa to 60.1 kPa. And the fabricated shape-memory scaffolds with various patterns and geometrics could be injected and applied as aorta scaffold.

Three-dimensional printing processes have been a cost-effective way to fabricate customized vascular grafts [103]. Savoji et al. [95] prepared poly(dimethyl itaconate-citric-octanediol) (PITCO) vascular tubes using prepolymers synthesized from dimethyl itaconate (DMI), triethyl citrate (TEC), and 1,8-octanediol (OD) through 3D printing (Figure 4C). The vascular tubes were formed within a short crosslinking time by UV irradiation. Their elastic modulus is in the range of 11–53 kPa, which varies by the component ratio and matches the mechanics of cardiac tissues. These vascular tubes could effectively support the adhesion and proliferation of umbilical vein endothelial cells, supporting cardiac tissue formation. These scaffolds were also shown to allow for exchanges of oxygen/nutrition and metabolic waste because the materials were semipermeable.

### 3.3. Neural Tissue Engineering

The treatment of traumatic injury of nerves has always been an important part of TE. The recovery function of adult peripheral nervous system is limited, making medical interventions essential for a better regeneration effect [104]. The conventional treatment approaches involve autologous nerve grafting, which is limited by such factors as the finite supply of autologous nerves, the need for a second surgical procedure, donor site complications, and immune reactions and complications. Furthermore, the clinical functional recovery rates of autologous nerve grafting are only around 80% [105]. In recent years, artificial nerve grafts have been reported and applied in nerve tissue engineering as the substitution of conventional nerve grafts. In addition to biocompatibility and biodegradability, the natural/synthesized materials designed for neural regeneration are expected to have properties/structures similar to that of the nervous systems, including permeability, biochemical activity, and architecture [106].

**Figure 5:**
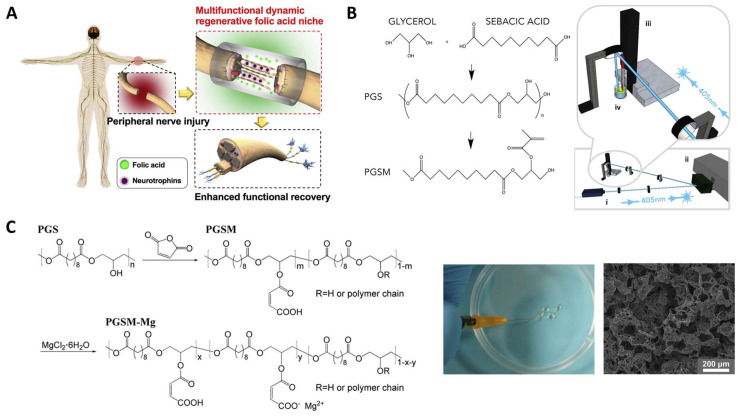
Nerve tissue engineering applications. (**A**) Folic acid (HDI-POC) on neural engineering [112]. Copyright 2018 Elsevier. (**B**) Biodegradable mAcr-PGS nerve guidance conduits and their fabrication method. (i) A 405 nm laser, (ii) a digital micromirror device, (iii) a motorized Zstage and (iv) liquid polymer. Reproduced with permission from Ref. [113]. Copyright 2018 Elsevier. (**C**) Injectable neuroactive Mg^2+^/PGSM hybrids. Reproduced with permission from Ref. [114]. Copyright 2019 Elsevier.

Functionalized polyester materials have been a recent focus in the field of neural TE. Various bioactive components were blended in the polyesters to impart electrochemical properties or emulate the biochemical activities of the nervous system. Wu et al. fabricated a conductive film of PGS-*co*-aniline crosslinked with HDI [107]. The conductive polyaniline enabled the films to conduct an electrical signal and promoted the therapy effect by inducing Schwann cells’ myelination and neurotrophin secretion. Calcium titanate (CaTiO_3_) was also mixed with PGS to achieve a similar effect [108]. In this research, the release of Ca^2+^ and its effect on axon outgrowth was studied. The conductive property of CaTiO_3_ was also important in the regeneration process. The mechanical properties of PGS elastomers were enhanced after incorporation of CaTiO_3_ which acted as reinforcing particles. As a result, Young’s modulus increased from 0.30 ± 0.05 MPa to 1.06 ± 0.08 MPa. Moreover, it has been demonstrated that calcium can promote the development of neural cells and foster functional connections between them, thus facilitating neural regeneration [109,110]. Ghafaralahi et al. reported polymeric matrixes that comprise PGS and PCL with various monomer ratios for nerve guidance conduit. Graphene nanosheets were added as a conductive filler to improve the mechanical properties and promote the biological properties [111]. PGS was shown to influence cellular behavior positively. Kim et al. [112] doped folic acid into HDI-POC to fabricate a nerve guidance conduit (fCUPE) (Figure 5A). With tailored scaffold mechanical properties and the regulating function of folic acid, the fCUPE conduit demonstrated a positive impact on the regeneration and functional recovery of the peripheral nervous system. Cell cytotoxicity and proliferation assays were performed to prove that the folic acid was non-toxic on cells. 

Unsaturated group modifications were also studied in neural TE. The methacrylated PGS (mAcr-PGS) nerve guidance conduits (NGCs) for neural regeneration were reported by Singh et al. [113]. The materials were fabricated and then postprocessed by laser cutting to achieve the tubes. These mAcr-PGS tubes displayed flexibility, resistance to kinking, and the capability to endure suturing, rendering them suitable for use in larger gap models (Figure 5B). Moreover, the in vitro analysis demonstrated the elongation and alignment of neurites within the NGC grooves, along with observed growth of both neuronal and glial cells. In the in vivo experiments, the mAcr-PGS conduits were found to facilitate axon regeneration, direct axonal growth, and did not lead to an increase in neuropathic pain. Sun et al. designed a hybrid of PGS-maleate and magnesium ions (PGSM-Mg) [114]. The Mg^2+^ interacted with polymer through the coordination bond. The obtained elastomers kept their soft nature and could be fabricated to elastic scaffolds with tailored porosity via 3D printing. The cell adhesion and proliferation of Schwann cells (SCs) were improved by the scaffolds (Figure 5C).

### 3.4. Bone Tissue Engineering

Over the last few decades, the incidence of bone injuries has significantly increased due to the aging population and rising cases of bone trauma and cancers. In response, bone tissue engineering (BTE) has been rapidly developed to provide biomaterials as substitutes for conventional bone grafts [115]. Materials studied for BTE included ceramics, polymers, bioactive drug, and their composites. Among these materials, osteoinductive biomaterials have shown promise. These biomaterials have the capability to induce bone formation by influencing the in vivo environment [116,117]. Osteoinductive ability has been found in several bioactive materials, such as calcium phosphate (CaPs) [118], hydroxyapatite (HA) [119], *β*-tricalcium phosphate (*β*-TCP) [120], and bioactive glasses [121]. Polyesters used in bone TE scaffolds include PCL, PLA, PGA, and their copolymers [122]. In general, scaffolds for BTE are expected to possess biocompatibility, biodegradability, osteoinductivity, and appropriate mechanical properties [123]. Incorporation of ceramic particles into polyester elastic scaffolds or the infusion of polyesters into ceramic scaffolds are common ways to improve the mechanical properties of scaffolds to make them suitable for bone regeneration. 

**Figure 6:**
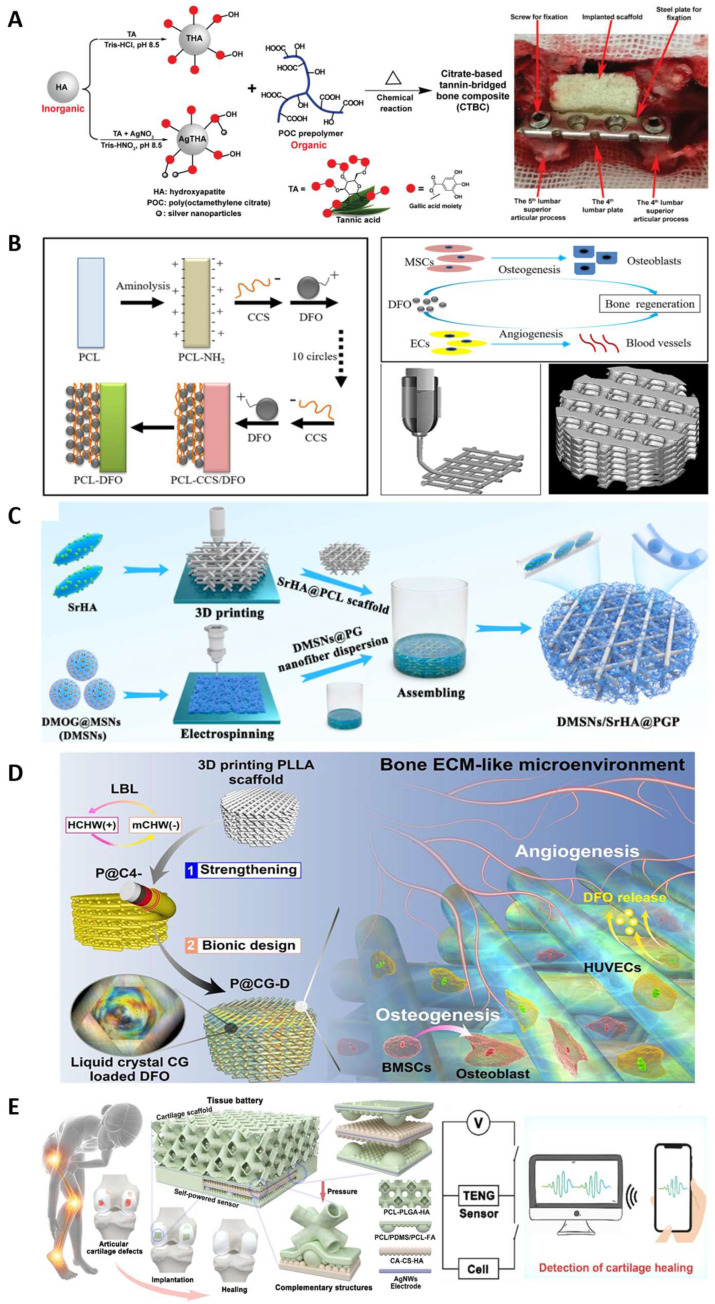
Bone tissue engineering applications. (**A**) Citrate-based bone composites for lumbar fusion. Reproduced with permission from Ref. [129]. Copyright 2020 Wiley. (**B**) Vascularized 3D printed scaffold delivering DFO. Reproduced with permission from Ref. [131]. Copyright 2019 Elsevier. (**C**) Three-dimensional printed biomimetic scaffold with promoted tissue infiltration and vascularization capacity. Reproduced with permission from Ref. [137]. Copyright 2023 American Chemical Society. (**D**) Bone ECM-like 3D printing scaffold for bone regeneration [138]. Copyright 2022 American Chemical Society. (**E**) Integrated cartilage therapy using tissue batteries of triboelectric nanogenerators. Reproduced with permission from Ref. [139]. Copyright 2023 Elsevier.

Most polyester elastomers developed for bone regeneration were combined with bioactive components. Citrate-based polyesters have shown significant promise in BTE applications, their multifunctional chemical properties make it possible to load various bioactive materials that promote osteogenesis [124,125,126]. Li et al. [127] developed a biodegradable poly(citrate-siloxane) (PCS) hybrid elastomer (PCS-SN) reinforced by silica nanoparticles (SNs). The SNs were evenly dispersed in the scaffolds. The weight ratio of SNs can be tuned to regulate the mechanical properties and biodegradability of the PCS-SN elastomers. The PCS-SN elastomers showed good histocompatibility, capable of promoting adhesion and proliferation of osteoblasts. Another PCS-based hybrid elastomer was incorporated with bioactive glass nanoparticles (BGNs), which contribute to biomineralization activities, facilitating bone tissue regeneration [128]. Guo et al. [129] designed a POC-based bone regeneration material coated with hydroxyapatite (HA) which prevents inorganic/organic phase separation (Figure 6A). As a natural bone component, HA plays an important role in lumber fusion, and it can enhance the mechanical properties of the materials. Tannic acid (TA) was coated to the surface of HA, and the silver nanoparticles interacted with the surface of HA to confer antimicrobial activity. The resulting tannin-bridged bone composites (CTBCs) based on POC display a significantly enhanced compression strength of up to 323.0 ± 21.3 MPa. These composites were biocompatible and could promote cell adhesion, proliferation, and biomineralization performance. Lumbar fusion model experiments on rabbits demonstrated the osteoconductive, osteoinductive, and bone regeneration promoting properties of CTBCs. By employing intrinsically fluorescent citric-based polyester synthesized from citric acid, 1,8-dioctanediol and L-Serine reacted with HA, Tan et al. [130] reported a bone putty (BPLP-Ser/HA) enabling the monitoring of material degradation kinetics. This composite showed mechanical properties (compressing strength and initial modulus) matching the early non-mineralized bone. The bone putty displayed malleability and could be press fitted into irregular defects with ease, demonstrating its handling properties that are on par with bone wax. This bone putty exhibited in vivo biocompatible and osteogenic potential in a rat calvaria model.

Over the past few decades, 3D printed scaffolds have garnered significant attention due to their distinctive three-dimensional porous structure, which provides the desired porosity and favorable mechanical properties. This allows them to closely mimic the natural trabecular bone [131]. Porous structures, being an essential element in scaffolds, provide a conducive microenvironment for cell adhesion, proliferation, differentiation, and biomineralization. Additionally, bioactive cues are necessary to create a synergistic microenvironment that accelerates the process of bone regeneration [132]. In recent research on bone regeneration, PCL and PLA have gained widespread use due to their excellent printability, the ability to control their mechanical properties, and their biodegradability [131,133,134]. Three-dimensional printed scaffolds incorporating bioactive materials for vascularity and osteoinduction are a common method in bone defect treatment, because bone regeneration is a continual and complex process, in which the angiogenesis and osteogenesis are tightly related to each other. Yan et al. [131] fabricated a PCL-based biodegradable scaffold which could deliver deferoxamine (DFO), an FDA approved iron chelator with the potential to promote vascularization and bone regeneration. The PCL scaffolds were fabricated by 3D printing and DFO was loaded via aminolysis of PCL surface and layer-by-layer assembly (Figure 6B). The compression strength of PCL-DFO was 2.7 ± 0.3 MPa, matching that of cancellous bones. The cell viability and proliferation tests revealed that the 3D printed PCL scaffold had good biocompatibility and DFO did not show a significant effect on cell growth. Further analysis of PCL scaffolds and PCL-DFO scaffolds indicated that the incorporation of DFO was essential for in vivo angiogenesis and osteogenesis, and the degradation profile of PCL-DFO scaffolds matched bone development and reconstruction. Three-dimensional printed scaffolds were also reported in patents. For example, Zhou et al. [135] described a polyester resin synthesized from α-ketoglutaric acid and 1,2-propanediol. Low temperature 3D printing was employed to fabricate a bone scaffold with an optimized pore size. Another polyester resin polypropylene fumarate (PPF) was synthesized to achieve a photocurable material which can be applied as bone regeneration scaffolds [136]. The viscosity of PPF was 24 Pa·s, which is higher than the general viscosity of polymer for photocuring 3D printing. Therefore, PPF can be dissolved in a solvent, then printed into a porous bone scaffold in 2 h. 

Dual-drug or multiple-drug delivery scaffolds are extensively sought after to promote bone regeneration, as the regeneration process is regulated by a variety of bioactive molecules [132]. Hybrid scaffold is a promising method to match the bone microenvironment, carry the cells, and deliver multiple bioactive agents [137,140,141,142]. Zhou et al. [137] developed a biomimetic scaffold by assembling short electrospun nanofibers containing mesoporous silica nanoparticles which were loaded with dimethyloxalylglycine (DMOG). This was combined with a 3D printed scaffold made of strontium (Sr) contained HA and PCL (SrHA-PCL) (Figure 6C). The compression modulus of SrHA-PCL scaffolds was 29.85 ± 4.75 MPa, and the mechanical stress was 1.87 ± 0.13 MPa. The degradation rate of electrospun nanofiber was faster than SrHA-PCL scaffold, facilitating the tailored release of DMOG and Sr ions. The scaffolds were proven to have biocompatibility and angiogenesis functions. This sequential release accelerated bone tissue growth and promoted vascularized bone regeneration. Liu et al. [138] reported a bone ECM-mimic 3D printing scaffold of PLLA (Figure 6D). The chitin whiskers were electrostatically self-assembled on the surface of PLLA via the layer-by layer method to strengthen the PLLA scaffold. Then, DFO was encapsulated in chitosan/chitin whiskers hydrogel, which exhibited an ECM-like liquid crystalline state and viscoelasticity. Thereafter, the hydrogel was introduced into the reinforced PLLA scaffold to create a bone ECM-mimic microenvironment. The obtained scaffold showed effective angiogenesis and osteogenesis promotion. PCL can also act as the coating on the 3D printed hydrogel scaffolds to form the core/shell hybrid scaffolds [132,143]. The hydrogel templates were fabricated by gelatin, alginate, chitosan or their mixtures. The degradation of the sacrificial hydrogel template is much faster than PCL, then a hollow channeled scaffold was obtained as there was core space left. These hollow channels serve as distinct architectural cues that promote bone formation and vascularization.

**Figure 7:**
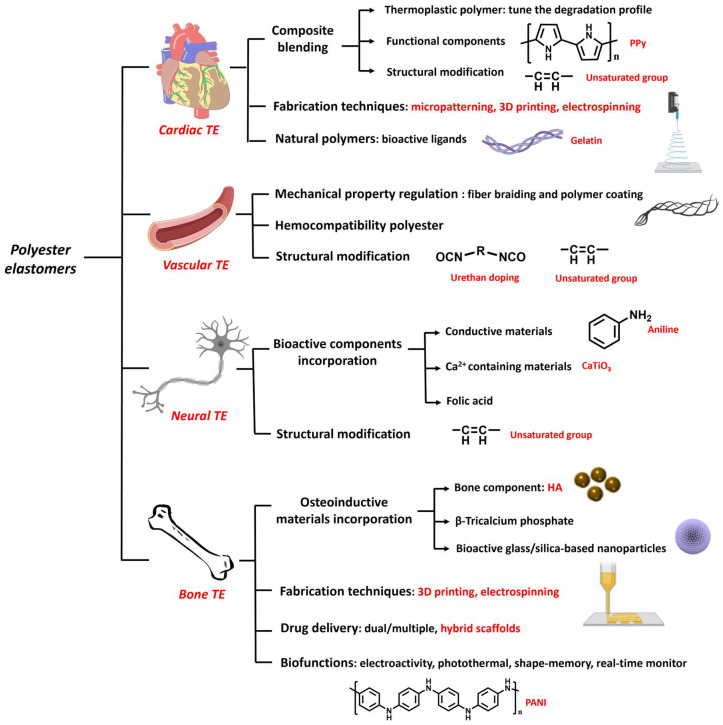
Schematic diagram of functionalized modified polyester elastomers for tissue engineering.

Another trend in recent bone regeneration research is introducing biofunctions, including electroactivity, conductivity [144], shape-memory property [145], and photothermal effects [146]. The real-time monitoring of regeneration states can be achieved by introducing a multi-convex triboelectric nanogenerator (TENG)-based sensor [139] (Figure 6E). For example, Zhang et al. reported conductive porous scaffolds by introducing camphor sulfonic acid-doped polyaniline (PANI) into HA/PLGA scaffolds [144]. The conductive scaffold was beneficial to osteogenesis and the PANI regulated the degradation behavior of the scaffold, preventing the severely compromised mechanical functionality caused by bulk erosion of PLGA (Figure 7). Most recently, Wang et al. [147] reported an optoelectronic composite scaffold that comprises silicon (Si) thin films with specific patterns and HA-collagen/PCL. The Si thin films can generate electrical signals and interact with the tissue when exposed to external light, regulating the cellular behavior. The HA-collagen/PCL constitutes the porous matrix that can topographically and mechanically support cell growth and differentiation. The Si films were tested and showed ideal biocompatibility. This biomimetic multiscale hierarchical architecture of the scaffold was biocompatible and found to improve bone regeneration efficiency.

### 3.5. Bioelectronics

There has been a growing interest in applying stimuli-responsive and highly elastic devices in implantable or wearable electronics and flexible sensors. The wearable sensors are expected to be biocompatible and have matching mechanical properties with the host tissues [148]. Therefore, the stretchability and compressibility of these elastomers are essential. Commercial elastomers (such as polyethylene terephthalate, polyimide, polyethylene, acrylonitrile butadiene styrene, polystyrene-ethylene-butylene-styrene, and polydimethylsiloxane) for wearable electronics often show high stiffness, which may lead to discomfort in touch [148,149,150]. Therefore, there has been a demand for the development of flexible bioelastomers including polyester elastomers. Traditional implantable bioelectronics for short-term applications have limitations such as inadequate mechanical properties, risks of infection, and a need for removal surgery after the complete treatment or monitoring [151]. To overcome the disadvantages of conventional implants, polyester elastomers have been developed due to their capacities to be dissolved or be degraded in situ. Recently, polyester elastomers developed for bioelectronic applications included PGS-based elastomers, POC-based elastomers, and PCL-based elastomers.

**Figure 8:**
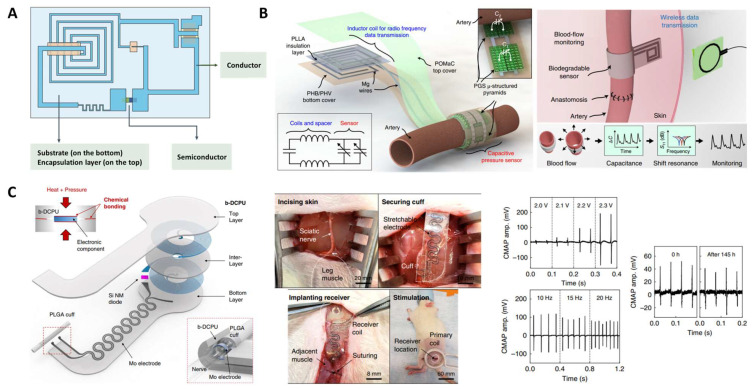
Implantable bioelectronics. (**A**) The essential components of implantable electronic devices degradable in vivo. Reproduced with permission from Ref. [152]. Copyright 2017 Springer Nature. (**B**) Soft multilayer electronics for arterial-pulse monitoring. Reproduced with permission from Ref. [153]. Copyright 2019 Springer Nature. (**C**) Bioresorbable electronic stimulators for neuromuscular regeneration. Reproduced with permission from Ref. [52]. Copyright 2020 Springer Nature.

To fabricate implantable electronics, insulating and biodegradable polyester elastomers were developed as an encapsulation layer, substrate, and dielectric material. In bioabsorbable electronics, the encapsulation layer shields the electrode from the external environment. The materials used in the encapsulation layer decide the degree of exposure of the electronics. The substrate provides a platform for microelectronic fabrication, while the dielectric materials endow functionality of the electronics [152] (Figure 8A). The degradation ratios of all these bioabsorable materials regulate the lifetime of the device. Boutry et al. [153] created a biodegradable pressure sensor that measures arterial blood flow with high sensitivity. The blood flow signal can be detected in both contact and non-contact manners by employing the fringe-field capacitor technology. This sensor comprised a micro-structured PGS dielectric layer in the pressure sensitive regions, POMaC and poly-hydroxybutyrate/polyhydroxyvalerate (PHB/PHV) packaging layers, and a PLLA spacer for the bilayer coils (Figure 8B). All the layers were ultrathin films and laminated together with a magnesium (Mg) electrode to fabricate the sensor. A dynamic covalent elastomer (b-DCPU) synthesized from PCL-triol and HDI was deployed by Choi et al. to serve as an encapsulating layer for a bioabsorbable wireless stimulation device [52]. The thermally activated dynamic bond exchange reactions enabled robust self-bonding between b-DCPU layers, resulting in good interfacial toughness between layers (Figure 8C). The device was employed for sciatic nerve stimulation for 30 days in a rat model, keeping its monitor sensitive and indicating the stable and long service life of the device. 

**Figure 9:**
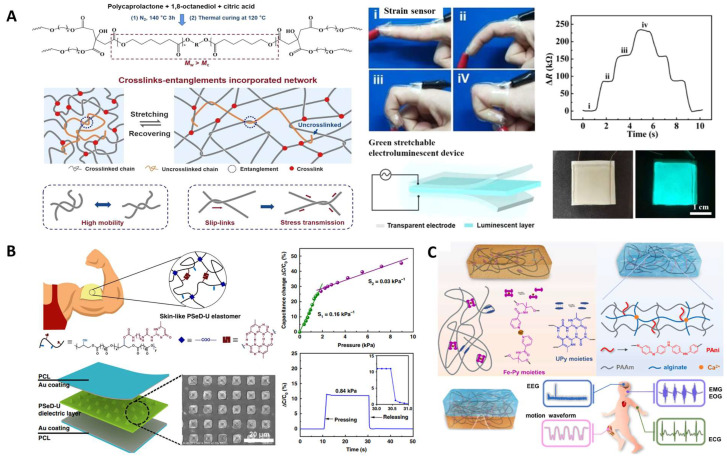
Wearable bioelectronics. (**A**) Elastomers with low hysteresis as strain sensors and electroluminescent devices. (i–iv) Monitor of movement of fingers. Reproduced with permission from Ref. [157]. Copyright 2022 American Chemical Society. (**B**) Skin-like elastomers for piezocapacitive pressure sensor. Reproduced with permission from Ref. [53]. Copyright 2020 Springer Nature. (**C**) Hybrid of elastomer and conductive hydrogel for flexible electronics. Reproduced with permission from Ref. [161]. Copyright 2022 Elsevier.

Polyester elastomers have been employed as the substrates of wearable biosensors due to their outstanding elastic properties. Conductive components such as metals [154], nanotubes [155], nanowires, nanosheets, conductive polymers like poly(ethylenedioxythiophene): poly(styrenesulfonate) (PEDOT:PSS) [156], ionic liquids [157], and semiconductive polymers [158] were incorporated into polyesters to improve their electrochemical properties. Sencadas et al. [159] designed a sensitive piezoresistive sensor that comprises elastomeric porous PGS. Multi-walled carbon nanotubes (MWCNTs) were incorporated to enhance its electromechanical performance. This flexible sensor is highly sensitive, capable of detecting pressure as low as 100 Pa within a response time of 20 ms. The foam structure of PGS was found to contribute to the high sensitivity of the sensor. PGS-urethane functionalized with PEDOT:PSS and CNTs was used to fabricate a strain sensor, which possessed both biocompatibility and biodegradability [155]. Hwang et al. [160] fabricated a skin sensor using POC substrates combined with biodegradable metal Mg to measure the biopotential and pH level. Chu et al. [157] fabricated strain sensors made of a biodegradable elastomer poly(1,8-octanediol-co-citrate-co-caprolactone) (POCL) incorporated with a conductive ionic liquid (IL) 1-ethyl-3-methylimidazolium bis(trifluoromethylsulfonyl) imide ([EMI]^+^ [TFSI]^−^) (Figure 9A). They designed the entangled flexible chains by using PCL diol with a molecular weight (*M_w_*) that is larger than its entanglement molecular weight (*M_c_*), facilitating the good resilience and low hysteresis of the sensor. The tensile strength of POCL was 0.20 MPa and the elongation was 770%, revealing suitable mechanical properties for epidermal electronics. POCL-based strain sensors showed a self-adhesive property, making it possible to be attached to the skin surface tightly, helping with their high sensitivity and fast response. 

Recent developments of polyester-based wearable biosensors also focused on the modification of polyester structures to achieve appropriate mechanical properties that better match those of human skin, and to endow a self-healing property to enhance the durability and reliability of the sensors [162,163]. Zhang et al. [164] designed poly(sebacoyl 1,6-hexamethylenedicarbamate diglyceride) (PSeHCD) elastomers using both chemical and physical crosslinking. The urethane units introduced hydrogen bonds as dynamic physical bonds which contribute to the self-healing property. These elastomers were coated with PEDOT:PSS in the fabrication of strain sensors that exhibited real-time signal outputs. Chen et al. [53] designed a poly(sebacoyl diglyceride) (PSeD)-graft-Upy (PseD-U) elastomer to be used in a dielectric layer in piezocapacitive pressure sensors (Figure 9B). The incorporation of Upy units provided effective energy dissipation through the sacrifice of hydrogen bonds. The PseD-U elastomers showed mechanical properties (Young’s modulus of 0.64 ± 0.10 MPa, tensile strength of 0.73 ± 0.10 MPa, elongation of 297 ± 16%) similar to those of human skin due to the combination of physical and covalent crosslinking [35]. Fabricated with micro-structured PseD-U elastomers and Au-coated PCL films, the piezocapacitive pressure sensors showed high pressure sensitivity and fast response, demonstrating that the PseD-U elastomers were appropriate substrate materials in wearable electronics. 

Elastomers have also been used in combination with conductive hydrogel in bioelectronic devices. Biomimetic multilayered structures, which are ubiquitous in human tissue, have provided inspirations for bioelectronics development [165]. To simulate the heterogenous structure of biological tissues in which different tissue layers vary in mechanical properties and biological functions, tough elastomers and stretchable hydrogels were integrated in advanced flexible electronics [166,167,168,169]. Generally, the modulus of the elastomers is tuned to match that of hydrogels, as the low-modulus hydrogels may cause signal noise due to the presence of motion artifacts. Liu et al. combined tough elastomers with conductive hydrogels to achieve a sensor with improved electronic signal that can be comparable with commercial Ag/AgCl electrodes in the detection of electrocardiogram (ECG) [161] (Table 2). The elastomers (CPU-Fe-Py-U) with high modulus and stretchability were synthetically based on poly(ε-caprolactone-co- DL-lactide)-glycerin (G-PLCL) polyester backbone. Upy was the physical crosslinker providing hydrogen bonding, and N-(1,3-dihydroxy-2-methylpropan-2-yl) pyridine-3-carboxamide was the coordination ligand to interact with Fe^3+^. The desirable mechanical properties were achieved by the cooperation of quadruple hydrogen bonding and coordination bonding, which could both tune the crosslinking density and flexibility of polymer chains. The resulting elastomer had tensile strength of 11.52 MPa and elongation of 1150%. The conductive hydrogel containing polyaniline (PANI) served as the sensitive component. This elastomer hydrogel hybrid structure sensor showed high sensitivity and fast response (10 ms) as a skin sensor (Figure 9C).

## 4. Conclusions and Perspectives

### 4.1. Conclusions

Polyester elastomers exhibit great potential in various biomedical applications including cardiac, vascular, neural, and bone tissue engineering and bioelectronics. Polyester materials are biocompatible and in vivo biodegradable, making them promising candidates in these biomedical applications. In recent years, there has been increasing interest in polyester elastomers with superior mechanical and biological properties that can be developed by exploring innovative polymer synthesis/modification methods and co-polymerization strategies. In addition, material processing technologies have been further developed to match the materials to the targeted applications. Moreover, polyester elastomers have generally been combined with bioactive materials to supply reactive sites, introduce a therapeutical effect, and/or to provide a biomimetic microenvironment to improve tissue regeneration. Overall, exploring polyester elastomers for biomedical treatment is becoming an attractive strategy in biomedical applications.

### 4.2. Perspectives

Despite the rapid progress in the development of polyester elastomers, there have been several aspects in this field that require further work and explorations. Firstly, there is still room for the structural design of polyester elastomers to achieve better performance and wider application. Further work on innovative synthetic strategies is necessary to endow multiple functionalities to the materials. Secondly, these properties such as shape memory and injectability of polyester elastomers need to be further explored because they exhibit great potential in providing an easy and minimally invasive surgery. The application of these properties can be expanded to cardiac repair patches, bioelectronics for in vivo monitors, or bone repair scaffolds. Thirdly, the fabrication method of hybrid scaffolds could be further improved. Similar to the implants, hybrid scaffolds that comprise polyester elastomers incorporated with bioactive components generally show better therapeutic effect than polyester elastomers. Exiting methods mostly involve mixing polyesters with bioactive components directly and fabricating the different layers separately, then assembling them together. Integrated processing methods are therefore demanded to simplify the fabrication process. Additionally, 3D fabrication methodologies are yet to be tuned to control the porosity and pore size of the 3D architecture when hybrid scaffolds are fabricated. As compared to other technologies (electrospinning, salt leaching), 3D printing allows for the preservation of cell viability and function, making it possible for in situ bioprinting. It is still a challenge to process the composites of polyester and the hydrogel/natural polymers with suitable mechanical properties and bioactivity. Hybrid scaffolds and multilayer elastomeric scaffolds have been widely studied because their designs simulate natural tissues. Nevertheless, the interactions between the polyester elastomers and the other layers were understudied. Benzophenone is a photo initiator reported to form chemical interactions between an elastomer and a hydrogel when the hybrid hydrogel/elastomer structure is UV crosslinked [145]. How to achieve a strong chemical or physical interaction between the elastomers and other hydrophilic components in the hybrid materials can be an interesting topic in further research to promote the development of bioelectronics. Finally, developing new biocompatible monomers and polyester elastomers with specific properties can be attractive, especially for some bio-based monomers. Bio-based polyester elastomers have proven their unique functionality in tissue engineering because scaffolds have been designed to assist in some metabolic functions such as citrate metabonegenic regulation that promote osteogenic differentiation [130,170]. Therefore, the exploration of novel bio-based polyester elastomers and their roles in providing physiological functions in tissue engineering will be a promising area.

## Figures and Tables

**Table 1:** Mechanical properties and degradation behaviors of representative polyester elastomers for biomedical applications.

Materials	Young’s Modulus (MPa)	Tensile Strength (MPa)	Elongation (%)	Degradation	Ref.
PLCL	19.6–95	17.2–26.6	388–1974	19% in 15 weeks in vivo 10% in 26 weeks, 50% in 52 weeks in vitro	[13,14,15,16]
PGCL	110–292.98	0.28–8	100–168	20–40% in 40 days in vitro	[17,18]
PGS	0.05–1.5	0.4–1.5	100–500	13% after 35 days in vitro	[19]
POC	0.42−16.4	0.35−6.1	100–265	20% after 28 days in vivo 100% after 15−68 weeks in vitro	[20,21]
P3HB	74.45−3500	1.3−554	3.8−26	<10% in 6 weeks in vivo	[22,23,24]
P4HB	0.1−670	2.3−70	10−1450	2–12 months in vivo	[25,26,27,28]
PCLF	3–7	0.5–17	230−800	—	[29,30,31]

**Table 2:** Recent progress of polyester elastomer for biomedical applications.

Applications		Backbone Polyester Materials	Function of Elastomer	Other Functional Materials	Ref.
Cardiac tissue engineering	Cardiac repair patch	PGS	elastic	PBS-DLA	[69]
		PGS	conductive film	PPy	[72]
		POMaC	adhesive	dopamine	[70]
		PICO	injectable		[75]
		PGS-*co*-aniline	conductive		[75]
		PCL, PGS	3D printing		[78]
		PGS	conductive	PPy, collagen	[76]
		PGSA-g-EG	injectable		[84]
Vascular tissue engineering	Vessel treatment requiring expanding	PGA, PGCL	elastic		[91]
	vascular implants	PGS-palmitic acid	elastic		[93]
		POC	antithrombus and endothelialization	ePTFE, atRA	[97]
		POMaC	elastic		[102]
	cardiovascular tissue regeneration	PITCO	3D printing		[95]
Nerve tissue engineering	Nerve repair	PGS	conductive	CaTiO_3_	[108]
		PCL, PGS	conductive	graphene nanosheets	[111]
		Folic acid-doped CUPE	regulation of cells		[112]
		Methacrylated PGS	elastic		[113]
		PGS-maleate	injectable	Mg^2+^	[114]
Bone tissue engineering	Bone tissue regeneration	PCS	elastic	silica nanoparticles	[127]
				bioactive glass	[128]
	Lumbar fusion	POC	elastic	HA, TA, Ag NPs	[129]
	Bone putty	BPLP-Ser	intrinsically fluorescent elastic scaffold	HA	[130]
	Vascularized bone regeneration	PCL	long-term scaffold	Strontium-HA, DMOG-silica nanoparticles	[137]
			channeled scaffold	sacrificial hydrogel	[132,143]
	Bone regeneration	PLGA	conductive scaffold	Sulfonic acid-doped PANI, HA	[144]
Bioelectronic	Semiconductor device	PCL	elastic matrix		
	Pressure sensor	PGS POMaC, PHB/PHV PLLA	dielectric layer packaging layer spacer		[153]
	Stimulation device	PCL	substrate and encapsulant		[52]
	Piezoresistive sensor	PGS	piezoresistive layer	CNTs	[159]
		PSeD-U	piezoresistive layer	Au	[53]
	Strain sensor	PGS-urethane	sensor layer	PEDOT: PSS-functionalized CNTs	[130]
		POCL	elastic matrix	[EMI]^+^[TFSI]^−^	[157]
		PSeHCD	elastic matrix	PEDOT: PSS	[156]
	Electronic device	G-PLCL	elastic layer with high modulus and stretchability	conductive hydrogel	[161]

## Data Availability

Not applicable.
